# Development and validation of a standardized double-blind, placebo-controlled food challenge matrix for raw hazelnuts

**DOI:** 10.1186/s13601-017-0181-8

**Published:** 2018-01-26

**Authors:** Marjolein Vandekerckhove, Bart Van Droogenbroeck, Marc De Loose, Katleen Coudijzer, Marc Coppens, Philippe Gevaert, Hilde Lapeere

**Affiliations:** 1 Technology and Food Science Unit, Research Institute for Agriculture, Fisheries, and Food (ILVO), Burg. Van Gansberghelaan 115, 9820 Merelbeke, Belgium; 2 Faculty of Medicine and Health Science, Department of Dermatology, Ghent University, De Pintelaan 185, 9000 Ghent, Belgium; 3 Faculty of Medicine and Health Science, Department of Anesthesiology and Perioperative Medicine, Ghent University, De Pintelaan 185, 9000 Ghent, Belgium; 4 Faculty of Medicine and Health Science, Department of Otorhinolaryngology, Ghent University, De Pintelaan 185, 9000 Ghent, Belgium

**Keywords:** Double-blind, Placebo-controlled food challenge, Hazelnut allergy, Diagnosis food allergy, Challenge matrix

## Abstract

**Background:**

Double-blind, placebo-controlled food challenge (DBPCFC) is considered the gold standard for food allergy diagnosis. However, this test is rarely performed routinely in clinical practice because of various practical issues, e.g. the lack of a standardized matrix preparation. The aim of this study was to develop and validate a convenient DBPCFC matrix, that can easily be implemented in daily clinical practice. The focus of this study was the blinding of hazelnuts, whereby the hazelnuts retained as much as possible their allergenicity and could be mixed homogenously in low-doses to the matrices.

**Methods:**

A basophil-activation test (BAT), microbial tests and an LC-MS/MS test were performed to assess respectively the allergenicity of the used hazelnuts, the microbial stability of the novel developed matrices and the homogeneity of the hazelnuts in the matrices. A sensory test was conducted to validate the blinding of the hazelnuts in the matrices. A pilot DBPCFC study included eight patients as proof of concept.

**Results:**

The BAT-test gave the first insights concerning the retained allergenicity of the hazelnuts. The microbial safety could be assured after 12 months of storage. Sufficient masking was assessed by several sensory tests. Homogeneous hazelnut distribution could be achieved for the different hazelnut concentrations. The DBPCFC’s results showed diverse allergic responders (from no reactions to distinct objective symptoms).

**Conclusion:**

A novel stable and validated DBPCFC matrix using raw hazelnuts has been developed that allows easy preparation in a standardized way for convenient use in daily clinical practice.

*Trial registration* EC Project number: EC/2015/0852; Date of registration: 13 Oct 2015; End date: 01 Feb 2017

**Electronic supplementary material:**

The online version of this article (10.1186/s13601-017-0181-8) contains supplementary material, which is available to authorized users.

## Background

The double-blind, placebo-controlled food challenge (DBPCFC) is considered the gold standard for food allergy diagnosis. This is however not common practice in the daily clinical setting due to several limitations, such as the lack of standardized preparation of the matrices [1]. One of the limiting factors of the DBPCFC is that the clinical staff must be able to prepare the matrices by themselves, so too labor-intensive preparations (e.g. baking, etc.) are not likely to be used in the routine.

However, from the clinical perspective, this test is useful to e.g. exclude/confirm a possible food allergy when other in vivo*/*in vitro tests are producing contradictory or inconclusive results, evaluate if patients have outgrown their allergy and to assess the severity of their reactions. For scientific reasons, it can be used to estimate the global prevalence of relevant food allergies, to assess the predictive value of other diagnostic methods and to determine specific threshold levels. However, proper validation of these matrices has only been performed in limited studies [2–5]. Further, low allergen dose matrices are needed to determine e.g. the minimum eliciting doses (MED’s) of the patients and to include safely severe allergic patients in this test [6]. As reported in 2014 by the Voluntary Incidental Trace Allergen Labelling 2.0 (VITAL) initiative, the MED in 1% of the hazelnut allergic population (ED_0.1_) is estimated to be 0.1 mg hazelnut proteins [6]. However, limited studies have yet been conducted to develop lower dose vehicles whereby the homogeneity was determined.

For hazelnuts, as far as we know, only a low-level matrix was developed using defatted hazelnuts (starting with 3 µg hazelnut proteins) [2]. However, defatting removes oil-body associated allergens which can also provoke severe allergic symptoms [7]. The high-fat content of hazelnuts (± 61% w/w) however makes homogenization and hence masking of the non-defatted nuts difficult.

This project focused on the development and validation of a novel, easy-to-use DBPCFC matrix for raw hazelnuts at low-level, which can be used in a standardized way in daily clinical practice and by multiple centers to allow comparison of test results. This study reports to our knowledge, the first validated matrix containing low doses of raw, non-defatted hazelnuts and no other major allergenic ingredients listed in the Regulation (EU) No. 1169/2011.

## Methods

### Preparation of the DBPCFC matrices

We developed a powder mix that results in a cold chocolate dessert after the addition of water. The ingredients are listed in Table 1. Blanched raw hazelnuts were ground in a RETSCH ZM200 mill (RETSCH, Düsseldorf, Germany) with a 0.75 mm sieve and afterward rolled with the Exakt 3-wals 80E (Exakt Technologies, Oklahoma, US) at 350 rotations/min. The protein content was determined using Kjeldahl analysis (according to ISO8968-2, conversion factor = 5.18). The rolled hazelnuts were packaged under vacuum conditions and stored in the dark at ambient temperatures (20 °C).

**Table 1:** Ingredients used for the DBPCFC matrix preparation

Added ingredients	Brand	Powder mix I (g/portion)	Powder mix II (g/portion)
Vanilla sugar^a^	Imperial	10.00	11.00
Brown sugar^a^	Candico	2.50	4.10
Stevia^a^	Pure Via	2.50	2.40
Cacao 100%^a^	Callebaut	8.20	10.70
Cold thickener (Quelli)^b^	Dawn	2.30	1.25
Sunflower Lecithin Sun 400 organic^c^	Lecico	0.30	0.30
Vanilla aroma^c^	Dawn	0.50	0.40
Lemon extract^d^	LorAnn Super-Strength Flavor	0.06	0.09
Cocos extract^d^	LorAnn Super-Strength Flavor	0.10	0.17
Speculaas spices^c^	J. S. Polak	0.28	0.53
Rice flakes Vanille^a^	Olvarit	2.80	2.80
Nutramigen AA^e^	Mead Johnson	5.50	6.50
Total		35.04	40.24

^a^No allergens were mentioned on the product information sheet

^b^Has precautionary labeling on the product information for gluten, eggs, lupine, milk (incl. lactose), soy

^c^Explicitly mentioned that it contains no allergens

^d^No allergens were mentioned on the product information sheet, labeled as gluten-free

^e^Is an amino acid-based formula for babies with a severe cow’s milk protein allergy or with multiple food allergies, it’s an extensively hydrolyzed formula by breaking down completely the present proteins in amino acids

The DBPCFC series were freshly prepared the day itself (± 2–4 h prior start of the challenge) (Table 2). For the verum vehicles first, a dilution series was prepared with suspended hazelnuts in lukewarm water (± 30 °C) (Table 2). Each dilution was shaken vigorously until the hazelnuts were fully suspended prior preparing the subsequent dilution. Each dilution (65 g) was added per portion of powder mix II (= 40.2 g for verum series 5–9; see Table 1) or powder mix I (= 35.0 g for verum series 1–4; see Table 1). To prepare the placebo series, 65 mg lukewarm water was added per portion of powder mix I. All the samples were shaken vigorously and set in at 4 °C for at least 10 min. Of these preparations, the respective portion sizes were served as listed in Table 2. Similar portion sizes were served in the placebo series as in the verum series.

**Table 2:** Preparation of the DBPCFC verum dilution series

A. Dilution series of the hazelnuts suspended in water to develop the verum series	


B. Dilution series of the hazelnut DBPCFC verum series							
Instructions for preparation/portion				Additional information			
Portion number	Dilution number (65 g)	Powder mix I (g)	Served portion size (g)	Dilution factor of served hazelnuts	Hazelnut (mg)/portion	Hazelnut proteins (mg)/portion	Ppm (mg/kg) hazelnuts
1	0	35.0	2.50	x	0	0	0
2	4	35.0	2.50	1/10	0.0370	0.005	14.8
3	3	35.0	2.50	1/10	0.3704	0.054	148.0
4	2	35.0	2.50	1/10	3.691	0.535	1476.9

Instructions for preparation/portion				Additional information			
Portion number	Dilution number (65 g)	Powder mix II (g)	Served portion size (g)	Dilution factor of served hazelnuts	Hazelnut (g)/portion	Hazelnut proteins (mg)/portion	Ppm (mg/kg) hazelnuts
5	1	40.2	1.34	1/3	0.04	5.37	27,721.3
6	1	40.2	4.01	1/3	0.11	16.11	27,721.3
7	1	40.2	12.02	1/3	0.33	48.33	27,721.3
8	1	40.2	36.07	1/3	1.00	145.00	27,721.3
9	1	40.2	108.22	1	3.00	435.00	27,721.3

### Assessment of microbial stability

To assess the microbial stability of the materials used, the a_w_-values and microbial constitution of both the powder mixes and the rolled hazelnuts were determined. The water activity (a_w_) was determined using the Benchtop Water Activity Meter (Pullman, WA, USA). Enumerations of *Escherichia coli* (AFNOR BRD-07/01-07/93 method), *Bacillus cereus* (ISO 7932) and detection of *Salmonella* (ISO 6579) were performed in the ISO 17025 accredited lab for food microbiology at the Belgian National Reference Lab at Research Institute for Agriculture, Fisheries and Food (ILVO, Melle, Belgium).

### Assessment of hazelnut allergenicity

To gain the first insights concerning the preserved allergenicity of the blanched rolled hazelnuts, a basophil activation test (= BAT) was performed with three selected hazelnut allergic patients having the following allergic reactions: oral allergy syndrome (= OAS), a late onset of mild urticaria and a severe systemic reaction. Details of the protocol are described in the Additional file 1: BAT protocol.

### Assessment of hazelnut homogeneity

LC-MS analysis was performed to confirm the absence of hazelnuts in the powder mixes and to confirm homogenous distribution of the hazelnuts in the different verum concentrations. Two prepared verum series were sampled and analyzed over two separate time periods. Details of the protocol are described in the Additional file 1: LC-MS/MS protocol.

### Sensory testing

Sensory tests were performed in two main phases to evaluate the masking of the hazelnuts (panelists were all > 20 years). Panelists with food allergies were not included in this test. In phase Ia and Ib, the tests were performed in the same sensory evaluation booths, by 30 and 31 panelists respectively, who were used to perform sensory tests. A combination of verum samples containing the highest hazelnut concentrations and placebo’s (3 and 2 samples per panelist in phase Ia and Ib, respectively) were given sequentially in different orders. After phase Ia, the test was repeated after minor adjustments of the matrices. Between each sample, water and crackers were given. After every tasting, they had to answer if the dessert contained hazelnuts or not, and if yes, by tasting or smelling. In phase II, the same test was performed as in phase Ib, with a consumer’s panel (91 panelists). All the tests were analyzed using the Chi^2^-test with 95% CI. When there was statistically no difference between the answers of the verum and placebo samples, the hazelnuts were considered sufficient blinded.

### DBPCFC pilot study

To validate the usability of the matrices in practice, eight DBPCFC’s were performed with patients having a mild hazelnut allergy as proof of concept. For patient details, see Additional file 1: Table S2. The study was approved by the Ethical Committee of the University Hospital (UH) Ghent, and all the patients signed an informed consent prior to the start of the test. Details of the protocol are described in the Additional file 1: DBPCFC protocol.

## Results

The rolled hazelnuts (protein content: 14.5% w/w) could be added to the matrices homogenously without leaving visible particulates.

The BAT assays resulted in different half maximal effective concentration values (EC_50_) for the different patients. For patients A, B and C the following EC50 values were recorded, respectively: 0.34 and 1.6 µg/mL and 0.32 ng/mL hazelnut proteins (see in the Additional file 1: Figure S1). Blood taken from a non-allergic donor gave no activation of the basophils.

The water activity (a_w_) of the powder mix and rolled hazelnuts were respectively 0.486 and 0.477. The microbial enumerations were for *E. coli* < 10.0 CFU/g, *Salmonella* sp. 0/25 g and *Bacillus cereus* < 10.0 CFU/g for all the analyzed matrices, even after 1 year of storage at ambient temperatures (20 °C) in the dark.

In addition, no hazelnuts could be detected in the powder mixes after the sensory tests. In Phase Ia (in the sensory lab of ILVO), no significant difference existed between the placebo and verum group (P = 0.290). No significant differences were found in smell nor taste between the verum and placebo samples in the group of panelists who detected hazelnuts (resp. P = 0.286 and 0.102). In phase Ib (in the same sensory lab), 48% (whereof 40% guessed) of the panel answered correctly for the verum samples and 29% (whereof 44% guessed) thought hazelnuts were present in the placebo matrices. No significant difference was found between the placebo and verum group (P = 0.096). Also for smell and taste, no difference was seen (resp. P = 0.354 and 0.208). Of the consumer’s panel, 30% (whereof 26% guessed) and 35% (whereof 30% guessed) detected hazelnut in the verum and placebo samples respectively, so no significant difference was shown (P = 0.263). Also for smell and taste, no difference was seen (P = 0.581 and 0.154). For more details regarding the results of the sensorial tests, we refer to Additional file 1: Table S1.

From the LC-MS/MS analysis, a linearity of R^2^ = 0.999 was obtained after analyzing the different verum series in duplicate, confirming the expected hazelnut presence (see Additional file 1: Figure S2). No hazelnuts were detected in the blanc powder mixes.

The DBPCFC outcomes are summarized in Table 3. During the pilot study, patients 1, 3 and 5 experienced very mild and subjective allergic symptoms at the highest hazelnut dose. Only patients 2 and 4 had distinct objective signs when performing the test. Patient 6 had both for placebo and verum subjective complaints, with similar symptoms of OAS. Patients 8 and 9 experienced no allergic reaction during the test. When results were negative (patients 8 and 9) or questionable (patients 1, 3 and 5), open provocation tests were done which were all positive. However, these reactions were very mild (for patients 3 and 5 e.g. only a mild tingling on the lips or a raw feeling in the throat was experienced after consuming 5 raw hazelnuts).

**Table 3:** DBPCFC results of the eight patients

Results challenge			
Patient no.	Dose	Signs and symptoms^a^	Test outcome
1	Verum 3–9	Very mild tingling on the lips	+/−; Only mild OAS symptoms; open provocation test needed
	Placebo 1–9	–	−
	Open provocation: ½ hazelnut	Mild tingling on tongue and lips	+/−
2	Verum 4–5	Tingling of the lips, Pruritus Mild urticaria/angioedema of the lips (11 min after verum 4)	+; Test is stopped after dose 5 because of inconvenient allergic complaints
	Placebo 1–9	–	−
3	Verum 9	Very mild tingling on the lips	+/−; Only mild OAS symptoms; Open provocation test needed
	Placebo 1–9	–	−
	Open provocation: 5 hazelnuts	Raw feeling in the throat	+
4	Verum 2–7 Verum 8–9	Pruritus, urticaria lesion in the neck Sneezing, itchy nose	+
	Placebo 1–9	–	−
5	Verum 9	Very mild tingling of the lips	+/−; Only mild OAS symptoms; Open provocation test needed
	Placebo 1–9	–	−
	Open provocation: 5 hazelnuts	Raw feeling in the throat	+
6	Verum 1–9	Very mild tingling of the lips	Verum and placebo challenges gave both mild OAS symptoms. This could be due to the anxiety of the patient during the challenges. Open provocation test is needed
	Verum 9	Gastrointestinal: Nausea	
	Placebo 1–9	Very mild tingling of the lips	
	Placebo 9	Gastrointestinal: Nausea	
	Open provocation: ½ hazelnut	Mild tingling of throat and tongue	+/−
7	Verum 1–9	–	–
	Placebo 1–9	–	–
	Open provocation: ½ hazelnut	Mild tingling of throat and tongue	+/−
8	Verum 1–9		
	Placebo 1–9		
	Open provocation: ½ hazelnut	Urticaria: discrete blister lower lip	+/−

^a^Symptoms and signs were assessed according to the standardized clinical parameters checklist described in the work of Sampson et al. [1]. OAS symptoms/signs were reported extra

+, positive test because of distinct objective signs during the challenge; +/−, results are uncertain because of subjective symptoms during the challenge; −, no reactions were observed during the challenge

## Discussion

The main purpose of this work was the development of a DBPCFC matrix for hazelnuts which had to fulfill several criteria like the sufficient masking of the hazelnuts and maintaining as much as possible the allergenicity. Further, easy logistics and preparation, long stability and sufficient palatability of the matrices are required. To include as much as possible different food allergic patients in this test, no other major allergenic food allergens listed in the Regulation (EU) No. 1169/2011 were used as ingredients during the recipe development of these matrices.

In this work, blanched hazelnuts (protein content: 14.5% w/w) were used because the dark testa is difficult to mask, both visually and in taste. For this reason, these were also used in other DBPCFC studies [4]. The nuts were kept at 100 °C for 8 min to remove this testa. Reduced allergenicity of the heat-labile hazelnut Cor a 1 and 2 allergens was only noticed at > 140 °C [8] but not much is known concerning the impact of blanching hazelnuts. Minor or no differences in allergenicity were seen in other blanched tree nuts, however [8]. For the three patients tested, basophil activation was shown, even for the least sensitive patient, presuming a retained allergenic potential of the blanched hazelnuts. However, further research should examine the significant impact of blanching on the hazelnut allergenicity.

To limit the potential impact of further processing on the hazelnut allergenicity, a cold matrix preparation was chosen. The fat content of the verum and placebo matrices were kept at < 25% of the total energy (resp. 13 and 19% of the total energy) so delayed absorption due to a high-fat content will be minimized [4]. The rolled hazelnuts could be mixed in the powder without leaving visible particulates.

The stability of the preparations was assessed by determining the a_w_-values and the microbial contamination. The a_w_-value can predict the growth of bacteria, yeast, and molds over time. For the hazelnuts and powder mix, these were low enough to inhibit bacterial and mold growth (resp. a_w_ ≥ 0.91 and 0.8). In addition, no relevant microbial spoilage was observed by organisms typically associated with confectionery-related food products with low water activity, like hazelnuts, after 1 year of storage at room temperature (20 °C), so an easy logistics of both the powder mixes and rolled hazelnuts can be guaranteed [9–11].

Instead of the most frequently applied triangle test [12], which focuses on differences between matrices whatever the attribution of difference is, the sensory test performed in this study was specifically adapted for its purpose [4], which was comparable with earlier performed sensory tests for DBPCFC matrix validation [3–5], whereby one placebo and verum sample were offered. In this test, the samples were offered sequentially instead of simultaneously, as done in the study of González-Mancebo et al. [5]. When small differences are not linked to the presence of the allergenic ingredient, the samples can be offered sequentially [5]. This represents how a DBPCFC is carried out in clinical practice, whereby the matrices will be given on separate days. We can conclude that considering the followed procedure and the results obtained, the hazelnuts were successfully blinded in our developed matrices. Consequently, the newly developed DBPCFC matrices will minimize the psychological placebo-effects for the patients and the healthcare professionals.

As a proof of concept, DBPCFC’s with eight patients were conducted in the clinical setting. The dosage schedule was a combination of semi-logarithmic and logarithmic increases, as performed in an earlier DBPCFC study in 2012 for hazelnuts [2], whereby similar protein portions were administered. This schedule was performed to achieve the top dose with acceptable increments in an acceptable period when starting with low-level protein portions.

The most sensitive patient had already subjective symptoms after the first dose and developed objective signs after the second hazelnut dose (corresponding to 5 and 54 µg of hazelnut proteins, respectively). These findings were comparable with those found earlier whereby 30 µg of hazelnut proteins was the lowest eliciting dose [2]. However, other factors, which were not included in this study, like exercise, alcohol consumption, asthma and anxiety might exacerbate the allergic reactions, so follow-up studies are necessary [13]. Further, the challenges were performed during September–November, and pollen-related food allergies can be worse during and just after pollen season (for birch pollen: ± middle of March until the middle of May) [14]. Different explanations could reason the absence of allergic symptoms for the two negative responders, like possible matrix/processing effects, too low doses of hazelnuts and the removing of the testa. Open provocation tests are therefore of utmost importance at the end of the test. However, psychological effects cannot be excluded then. Since patients with anaphylaxis and children were excluded from this DBPCFC study, further research should be conducted including younger and more severe allergic patients. During the challenges of this work, the patients nor health care professionals could discover the verum series. Most of the patients found the deserts easy to consume.

## Conclusion

To conclude, this study reports the production and validation of a novel DBPCFC matrix for raw hazelnuts at low doses, in a format that allows easy logistics and implementation in the common clinical setting.

## Additional file

**Additional file 1: Table S1.** Sensory test results of phase I and phase II with semi-trained panel and consumers panel**. Table S2.** Clinical parameters of the hazelnut allergic patients**. Figure S1.** Trend curves of the BAT-responses towards different hazelnut protein concentrations of patients A (measured twice, A1 and A2), B and C. EC_50_ = Half maximal effective concentration/ Dose = protein concentration: µg/mL (patient B) and ng/mL (patient C)**. Figure S2.** LC-MS/MS results of the verum dilution series (Hazelnut peptide (R)INTVNSNTLPVLR(W) m/z 721.3 → 1013.7). Top: Standard curve of the verum dilution series. Down: Chromatographic peak of hazelnut peptide.

## Abbreviations

DBPCFC: double-blind, placebo-controlled food challenge
MED: minimal eliciting dose
VITAL: voluntary incidental trace allergen labelling
BAT: basophil activation test
LC-MS: liquid-chromatography mass-spectrometry
