# Supplementary material for: Development and validation of a standardized double-blind, placebo-controlled food challenge matrix for raw hazelnuts
Source: Clin Transl Allergy. 2018 Jan 26;8:3. doi: 10.1186/s13601-017-0181-8 (PMC5785854; doi:10.1186/s13601-017-0181-8)
Supplement: Supplementary file 1 — Additional file 1: Table S1. Sensory test results of phase I and phase II with semi-trained panel and consumers panel. Table S2. Clinical parameters of the hazelnut allergic patients. Figure S1. Trend curves of the BAT-responses towards different hazelnut protein concentrations of patients A (measured twice, A1 and A2), B and C. EC50 = Half maximal effective concentration/ Dose = protein concentration: µg/mL (patient B) and ng/mL (patient C). Figure S2. LC-MS/MS results of the verum dilution series (Hazelnut peptide (R)INTVNSNTLPVLR(W) m/z 721.3 → 1013.7). Top: Standard curve of the verum dilution series. Down: Chromatographic peak of hazelnut peptide. [file 13601_2017_181_MOESM1_ESM.docx]

# **SUPPORTIVE INFORMATION**

**Development and validation of a standardized double-blind, placebo-controlled food challenge matrix for raw hazelnuts**

**AUTHORS**

Vandekerckhove Marjolein^1^, MSD (marjolein.vandekerckhove@ilvo.vlaanderen.be)

Bart Van Droogenbroeck, Dr. Ir. ([bart.vandroogenbroeck@ilvo.vlaanderen.be](mailto:bart.vandroogenbroeck@ilvo.vlaanderen.be))

Marc De Loose, Prof. Dr. ([marc.deloose@ilvo.vlaanderen.be](mailto:marc.deloose@ilvo.vlaanderen.be))

Marc Coppens, Prof. Dr. ([marc.coppens@uzgent.be](mailto:marc.coppens@uzgent.be))

Katleen Coudijzer, Dr. ([katleen.coudijzer@ilvo.vlaanderen.be](mailto:katleen.coudijzer@ilvo.vlaanderen.be))

Philippe Gevaert, Prof. Dr. (philippe.gevaert@ugent.be)

Hilde Lapeere, Prof. Dr. ([hilde.lapeere@uGent.be](mailto:hilde.lapeere@uGent.be))

**SUPPORTIVE INFORMATION: METHODS**

# **BAT protocol**

Proteins were extracted as described by Platteau et al (E1), with minor modifications. At first, the hazelnuts were extracted by adding 4 mL PBS (Sigma-Aldrich, St. Louis, MO, USA; pH 7.4), which included a mix of protease inhibitors, to 0.4 g of sample (10 vol w/v). The mix of protease inhibitors included 2 mM ethylenediaminetetraacetic acid, 5 mM diethyldithiocarbamate, 0.5 mM benzamidinehydrochloride and 0.2 mM phenylmethylsulfonefluoride (Sigma-Aldrich, St. Louis, MO, USA). After shaking ON at 4°C, the samples were centrifuged for 1 hour at 4°C and 4000 x g. The supernatant was collected and filtered through 0.22 µm filters (Millipore, Billerica, MA, USA). The protein concentration was measured with the NanoDropTM spectrophotometer ND-1000 (Thermo Fisher Scientific, Merelbeke, Belgium) and dried with a nitrogen flux at 30°C. A standard series was prepared ranging from 1 mg/mL to 64 pg/mL using the stimulation buffer of the Flow CAST^®^ BAT kit. This Flow CAST® BAT kit (BÜHLMANN Laboratories AG, Schönenbuch/Basel, Germany) was performed according to the manufacturer instructions. Blood was collected using K_2_EDTA tubes (BD, Erembodegem, Belgium) and tested within two hours of sampling from three selected hazelnut allergic patients with different histories. The activated basophils were detected with the FACSCanto™ II System (BD, Erembodegem, Belgium) and analyzed with the CellQuest Pro software (BD). The flow cytometer was equipped to detect Forward Scatter (FSC), Side Scatter (SSC) and the two fluorochromes FITC and PE as described in the manual of the Flow CAST^®^ BAT kit. The CCR-3-PE gate was set at 600 counts (= number of basophils) to stop the acquisition.

# **LC-MS/MS protocol**

## **Reagents and chemicals LC-MS/MS**

The ureum was purchased from Amersham Biosciences (Uppsala, Sweden). The iodoacetamide was from G Biosciences (St. Louis, MO, USA). The reagents tris(hydroxymethyl)-aminomethane (TRIS, pH 7.8), Potassium chloride (KCl), ethylenediaminetetraacetic acid (EDTA) and dithiothreitol (DTT) were purchased from Merck (Darmstadt, Germany). Acetonitrile (ACN), methanol (MeOH), chloroform (all ULC-MS grade) and formic acid (FA) were purchased from Biosolve B.V. (Valkenswaard, the Netherlands) and trifluoroacetic acid (TFA) from Thermo Scientific (Waltham, MA, USA). Sep-Pak Vac 1cc C_18_ SPE reversed phases columns were from Waters (Milford, MA, USA) and 0.22 µm filters from Millipore (Billerica, MA, USA). The used water was MilliQ H_2_O (Billerica, MA, USA).

## **LC-MS/MS protocol**

The verum series were sampled and analyzed over two separate times with two different prepared challenge series. To all the samples of the verum dilution series, 15 mL homogenization buffer was added to 2.5 g of the samples, shaken overnight by 4°C and extracted as described by Minkof et al (E2). The digestion was done according to Costa et al (E3) with minor modifications. To the extracted proteins, 1 mL of digestion buffer was added. Next, the he following compounds were added sequentially: 50 µL 200 mM DTT, 200 µL 200 mM iodoacetamide and 200 µL 200 mM DTT were added and gently shaken for 1 hour between each step in the dark. Before tryptic digestion, the protein content was determined with the nanodrop ND-1000 (Thermo Fisher Scientific, Merelbeke, Belgium). The trypsin was added in 1:50 ratio to 6 mL (20.6 +/- 1.6 mg proteins for the diluted portions 1 – 4 and 47.3 +/- 0.2 mg proteins for the undiluted portion) of the reduced and alkylated proteins. After overnight incubation, the samples were cleaned with the 0.22 µm filters and desalted with the Sep-Pak Vac 1cc C_18_ SPE reversed phases columns (as described in the manual). As washing and elution buffers, resp. 1 mL of 2 % ACN + 0.1 % TFA and 2 mL 70 % ACN + 0.1 % TFA was used. The samples were dried under nitrogen flow at 40°C and resuspended in 500 µL Tris (pH 7.8) prior extra filtering through 0.22 µm filters.

## **UHPLC-parameters**

To separate the peptides, an Acquity^TM^ Ultra Performance LC system from Waters (Milford, MA, USA) with an Acquity^TM^ UPLC BEH_300_ C_18_ reversed phase column (Waters, 300 Å, 1.7 µm, 2.1 x 150 mm) and an Acquity^TM^ UPLC BEH_300_ C_18_ VanGuard pre-column (Waters, 300 Å, 1.7 µm, 1 x 5 mm) attached upstream was used. As mobile phases, mobile phase A (H_2_O and 0.1 % FA) and mobile phase B (ACN and 0.1 % FA) were used. As flow rate and column temperature, 0.2 mL/min and 40°C was respectively set. A linear gradient was set, starting from 99 % A + 1 % B to go to 60 % A + 40 % B in 60 min. A rinsing step was set, with 15 % A and 85 % B for 5 min. To return to the initial equilibrium, 15 min of the initial composition was retained. The injection volume was set to 5 µL and the samples temperature to 10°C.

## **MS/MS-parameters**

A tandem quadrupole MS (Xevo TQ-S; Waters) was used in parallel with the UHPLC separation as described earlier. Instrument configuration parameters were: ESI^+^, cone voltage 30 V, capillary voltage 3.20 kV, source temperature 130°C, desolvation temperature 350°C. Function parameters were: automatic cycle time (sec), span 0.2 Da, retention time (RT) was dependent of the selected peptide with an RT window of +/- 2 min, m/z-values of precursor-fragment transitions. CE-values for each fragment ion were peptide dependent. Following hazelnut specific peptides were detected: (R)INTVNSNTLPVLR(W) (RT = 29) with m/z precursor = 721.3 and m/z fragment ions = 392.1 (b3, CE 25, qualifier ion) and 1013.7 (y9, CE 25, quantifier ion) and (R)LNALEPTNR(I) (RT = 18) with m/z precursor =514.6 and m/z fragment ions = 299.3 (b3, CE 15, qualifier ion) and 487.2 7 (y5, CE 15, quantifier ion). To analyze results, peak areas of the detected peptides were integrated using the MassLynx v4.1 software.

# **DBPCFC protocol**

Patients with a history of anaphylaxis were excluded from the test. Anti-histamines and beta-blockers were excluded respectively 7 and 3 days prior to the challenge. Patients were sober before starting the test. The study was approved by the Ethical Committee of the University Hospital (UH) Ghent, and all the patients signed an informed consent prior to the start of the test. The challenges were conducted in the Ambulatory Surgery Unit of the University Hospital Ghent and supervised by a dermatologist experienced in provocation tests and a specialist in Anesthesia and Resuscitation. Before onset of the test, the general condition and vital parameters of the patients were assessed. An intravenous line was administered and resuscitation drugs were prepared. Also, a skin prick test (SPT) with raw hazelnuts was done and blood was taken to perform Phadia ImmunoCAP (Thermo Fisher Scientific, Ghent, Belgium) with specific hazelnut allergens (Total IgE, Cor a 1, Cor a 8 and Cor a 9). The verum and placebo series were given in different orders on separate days with a 20-min interval between each dose. During the challenge, the clinical parameters (allergic signs and symptoms) were scored as listed previously (1). The test was ended when patients had distinct signs and/or symptom, considered the allergic reactions to annoying to continue or couldn’t finish the dessert. When results were doubtful or negative, an open provocation test with raw hazelnuts was performed.

Further, it should be mentioned that the challenges were performed during September - November, and pollen-related food allergies can be worse during and just after pollen season (for birch pollen: +/- middle of March until middle of May). In our opinion, this parameter should be mentioned in the studies performing DBPCFC’s.

# **References**

E.1. Platteau CMF, Bridts CH, Daeseleire EA, De Loose MR, Ebo DG, Taverniers IV. Comparison and Functional Evaluation of the Allergenicity of Different Hazelnut (Corylus avellana) Protein Extracts. *Food Anal Methods* 2010;**3:**382-388.

E.2. Minkoff BB, Burch HL, Sussman MR. A Pipeline for 15N Metabolic Labeling and Phosphoproteome Analysis in Arabidopsis thaliana. In: Sanchez-Serrano JJ, Salinas J, editors. Arabidopsis Protocols. Totowa, NJ: Humana Press, 2014; 353-379.

E.3. Costa J, Ansari P, Mafra I, Oliveira MB, Baumgartner S. Assessing hazelnut allergens by protein- and DNA-based approaches: LC-MS/MS, ELISA and real-time PCR. *Anal Bioanal Chem* **2014**;406:2581-2590.

**SUPPORTIVE INFORMATION: TABLES**

**Table S1.** Results of sensory test, phase I and phase II.

|  | | | **PHASE I a** | | | **PHASE Ib** | | | **PHASE II** | | |
| --- | --- | --- | --- | --- | --- | --- | --- | --- | --- | --- | --- |
|  | **MATRIX** |  | **TASTING** | | **TOTAL** | **TASTING** | | **TOTAL** | **TASTING** | | **TOTAL** |
|  |  |  | **Detect hazelnut** | **Did not detect hazelnut** |  | **Detect hazelnut** | **Did not detect hazelnut** |  | **Detect hazelnut** | **Did not detect hazelnut** |  |
| **DESSERT** | VERUM | Count | 18 | 12 | 30 | 15 | 16 | 31 | 27 | 64 | 91 |
|  |  | Expected Count | 19,7 | 10,3 | 30 | 12 | 19 | 31 | 29,5 | 61,5 | 91 |
|  | PLACEBO | Count | 41 | 19 | 60 | 9 | 22 | 31 | 32 | 59 | 91 |
|  |  | Expected Count | 39,3 | 20,7 | 60 | 12 | 19 | 31 | 29,5 | 61,5 | 91 |
| **TOTAL** |  | Count | 59 | 31 | 90 | 24 | 38 | 62 | 59 | 123 | 182 |
|  |  | Expected Count | 59 | 31 | 90 | 24 | 38 | 62 | 59 | 123 | 182 |

# **Table S2.** Clinical parameters of the hazelnut allergic patients

| **Patient nr.** | **Age** | **Gender** | **Type of hazelnut allergic reaction** | **SPT (mm)** | **Total IgE (kU_A_/L)** | **IgE Cor a 1 (kU_A_/L)** | **Inhaled allergies ^A^** | **Other food allergies ^B^** | **Other allergies** | **Asthma** |
| --- | --- | --- | --- | --- | --- | --- | --- | --- | --- | --- |
| 1 | 67 | F | OAS | 5 | 19.9 | 1.44 | 1 – 3 | 1 – 4 | Penicilline | YES |
| 2 | 52 | F | OAS | 3 | 63.7 | 97.9 | 1 – 5 | 1, 5 - 7 | Ibuprofen | NO |
| 3 | 61 | M | OAS | 4 | 381 | 10.4 | 1 – 6 8 – 9 | 8 | Contrast fluid | NO |
| 4 | 34 | F | OAS | 3 | 605.9 | 32 | 1 – 8 | All raw vegetables and fruits, except for pine- apple, cucumber and lettuce | Penicilline | YES |
| 5 | 18 | F | OAS, inhaled, contact | 7 | NA | > 100 | 1 – 3 | 9 | NO | NO |
| 6 | 67 | M | OAS | 5 | 250.9 | 2,56 | 1 – 5, 8 – 9 | NO | NO | NO |
| 7 | 25 | F | OAS | 25 | 44.1 | 5.35 | 1 – 3, 6, 8 – 10 | 10 - 12 | NO | YES |
| 8 | 55 | F | OAS | 20 |  | 2.56 | 2, 8, 9 | 1, 2, 4, 5, 12 | NO | YES |

^A^ Inhalant allergies towards: 1 = alder, 2 = birch, 3 = filbert, 4 = dust mite, 5 = flour mite, 6 = grass, 7 = *Artemisia*, 8 = cat, 9 = dog, 10 = *Alternaria*

^B^ Food allergies towards: 1 = apple, 2 = peach, 3 = chicory, 4 = pear, 5 = cherry, 6 = pine apple, 7 = stone fruit, 8 = soy, 9 = most fruit species except pineapple, 10 = celery, 11 = artichoke, 12 = kiwi, 13 = cherry.

NA = Not analyzed

SPT = Skin Prick Test

**Figure S1.** Trend curves of the BAT-responses towards different hazelnut protein concentrations of patients A (measured twice, A1 and A2), B and C. EC_50_ = Half maximal effective concentration/ Dose = protein concentration: µg/mL (patient B) and ng/mL (patient C).


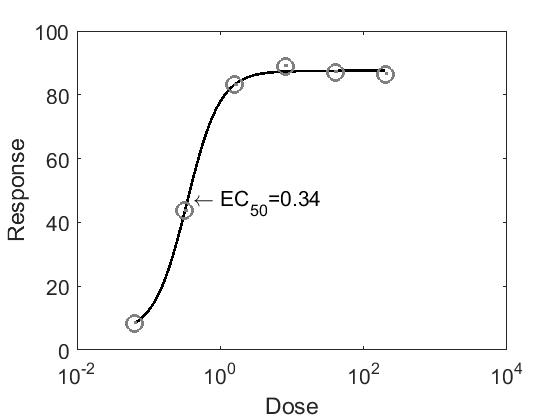

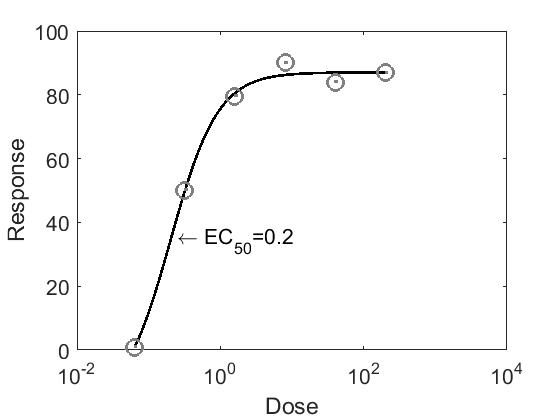


A.1

A.2


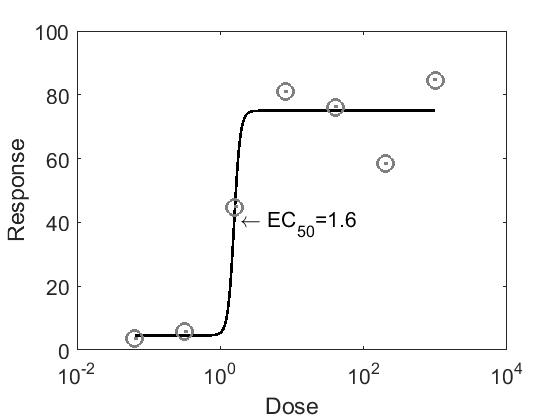

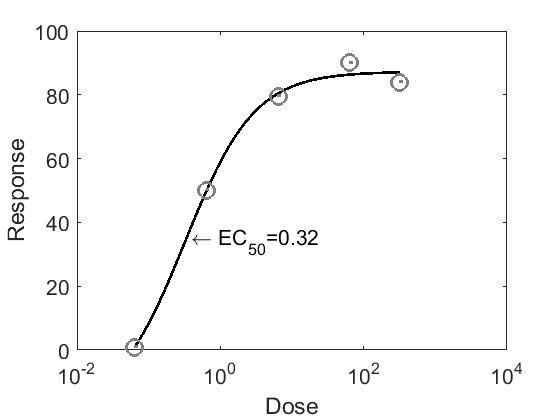


B

C

# Patient A, B and C experiences respectively oral allergy syndrome (OAS), a late onset of mild urticaria and a severe systemic reaction after consuming hazelnuts. Blood taken from a non-allergic donor gave no activation of the basophils.

**Figure S2.** LC-MS/MS results of the verum dilution series (Hazelnut peptide (R)INTVNSNTLPVLR(W) m/z 721.3 🡪 1013.7).

Top: Standard curve of the verum dilution series. Bottom: Chromatographic peak of hazelnut peptide


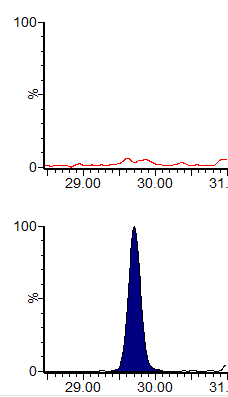


**14.8 ppm** hazelnut

(verum portion 2)

**0 ppm** hazelnut

(powder mixes)
